# Development of a novel score model to predict hyperinflammation in COVID-19 as a forecast of optimal steroid administration timing

**DOI:** 10.3389/fmed.2022.935255

**Published:** 2022-08-09

**Authors:** Yuichiro Takeshita, Jiro Terada, Yasutaka Hirasawa, Taku Kinoshita, Hiroshi Tajima, Ken Koshikawa, Toru Kinouchi, Yuri Isaka, Yu Shionoya, Atsushi Fujikawa, Yasuyuki Kato, Yasuo To, Yuji Tada, Kenji Tsushima

**Affiliations:** ^1^Department of Pulmonary Medicine, International University of Health and Welfare Narita Hospital, Narita, Japan; ^2^Department of Respirology, Graduate School of Medicine, Chiba University, Chiba, Japan; ^3^Department of Infectious Disease, International University of Health and Welfare Narita Hospital, Narita, Japan

**Keywords:** COVID-19, cytokine storm, hyper-inflammation, predicting score, corticosteroid

## Abstract

**Objectives:**

This study aims to create and validate a useful score system predicting the hyper-inflammatory conditions of COVID-19, by comparing it with the modified H-score.

**Methods:**

A total of 98 patients with pneumonia (without oxygen therapy) who received initial administration of casirivimab/imdevimab or remdesivir were included in the study. The enrolled patients were divided into two groups: patients who required corticosteroid due to deterioration of pneumonia, assessed by chest X-ray or CT or respiratory failure, and those who did not, and clinical parameters were compared.

**Results:**

Significant differences were detected in respiratory rate, breaths/min, SpO_2_, body temperature, AST, LDH, ferritin, and IFN-λ3 between the two groups. Based on the data, we created a corticosteroid requirement score: (1) the duration of symptom onset to treatment initiation ≥ 7 d, (2) the respiratory rate ≥ 22 breaths/min, (3) the SpO_2_ ≤ 95%, (4) BT ≥ 38.5°C, (5) AST levels ≥ 40 U/L, (6) LDH levels ≥ 340 U/L, (7) ferritin levels ≥ 800 ng/mL, and (8) IFN-λ3 levels ≥ 20 pg/mL. These were set as parameters of the steroid predicting score. Results showed that the area under the curve (AUC) of the steroid predicting score (AUC: 0.792, 95%CI: 0.698–0.886) was significantly higher than that of the modified H-score (AUC: 0.633, 95%CI: 0.502–0.764).

**Conclusion:**

The steroid predicting score may be useful to predict the requirement of corticosteroid therapy in patients with COVID-19. The data may provide important information to facilitate a prospective study on a larger scale in this field.

## Introduction

Numerous studies have shown that cytokine storms, a state of systemic hyper-inflammation, are among the most characteristic pathophysiologies of the severe coronavirus disease 2019 (COVID-19) (1, 2). According to the National Cancer Institute, a cytokine storm is a severe immune response that occurs when cytokines are excessively released into the blood, triggered by various causes, such as infectious diseases, autoimmune diseases, drug treatments, and malignant diseases (3). During COVID-19, the immune response begins with the local immune system and then progresses to the systemic immune system. Following viral infection in host tissues such as the lung, local innate and adaptive immunity is activated. These local immune systems trigger pro-inflammatory cytokines that develop into a systemic immune response (4). During cytokine storms, the overproduction of pro-inflammatory cytokines, such as tumor necrosis factor-alpha (TNF-α), interleukin-1 (IL-1), and interleukin-6 (IL-6) can result in systemic hyper-inflammatory responses, vascular hyper-permeability, and in rare cases, acute respiratory distress syndrome (ARDS), multiple organ failure, and death (1, 2). Thus, it is crucial in clinical practice to predict systematic immune responses (cytokine storms) in the early stages to prevent COVID-19 lung deterioration.

Corticosteroids are one of the most effective treatments for suppressing hyper-inflammatory immune responses in COVID-19 (5). Corticosteroid administration can suppress immune activation and reduce viral clearance, if initiated at the appropriate time (6). To prevent systemic inflammation in viral diseases, the following strategies are largely recommended: (1) reducing viral entry and replication by targeting critical components of these viral activities and (2) suppressing virus-induced inflammation by interfering with relevant host immune pathways. Among these, the latter approach corresponds to corticosteroids (4). Therefore, we hypothesize that if corticosteroid administration can be predicted in advance, it will be easier to determine whether hospitalization is required.

It has been reported that administering corticosteroids too early in the phase of COVID-19 lowers the inflammatory status and disease severity, increasing the viral load and causing harm to these patients (6). Thus, a scoring system that can predict hyper-inflammation rates requiring corticosteroid administration is required. In addition, whether or not any predicting score for the hyper-inflammation of COVID-19 exists remains an unanswered question. Furthermore, although the H-score is considered close to the score for predicting the systemic hyper-inflammatory status of COVID-19, it is considered inappropriate to be applied to COVID-19 because the cut-off value of the H-score for ferritin and fever is too high. Moreover, bone marrow biopsy findings need to be incorporated into the score. Notwithstanding, some studies have reported that the H-score may be valuable in COVID-19 prediction if modified (7–9).

Based on these facts, this study examined patients with COVID-19 hospitalized due to moderate pneumonia without oxygen administration. We also observed the characteristics of patients who were resistant to standard treatments and required corticosteroids because of worsening pneumonia (i.e., hyper-inflammation). Subsequently, we created a corticosteroid prediction score and examined the validity of this scoring system by comparing it to the H-score.

## Materials and methods

### Study design and patients

**Figure 1:**
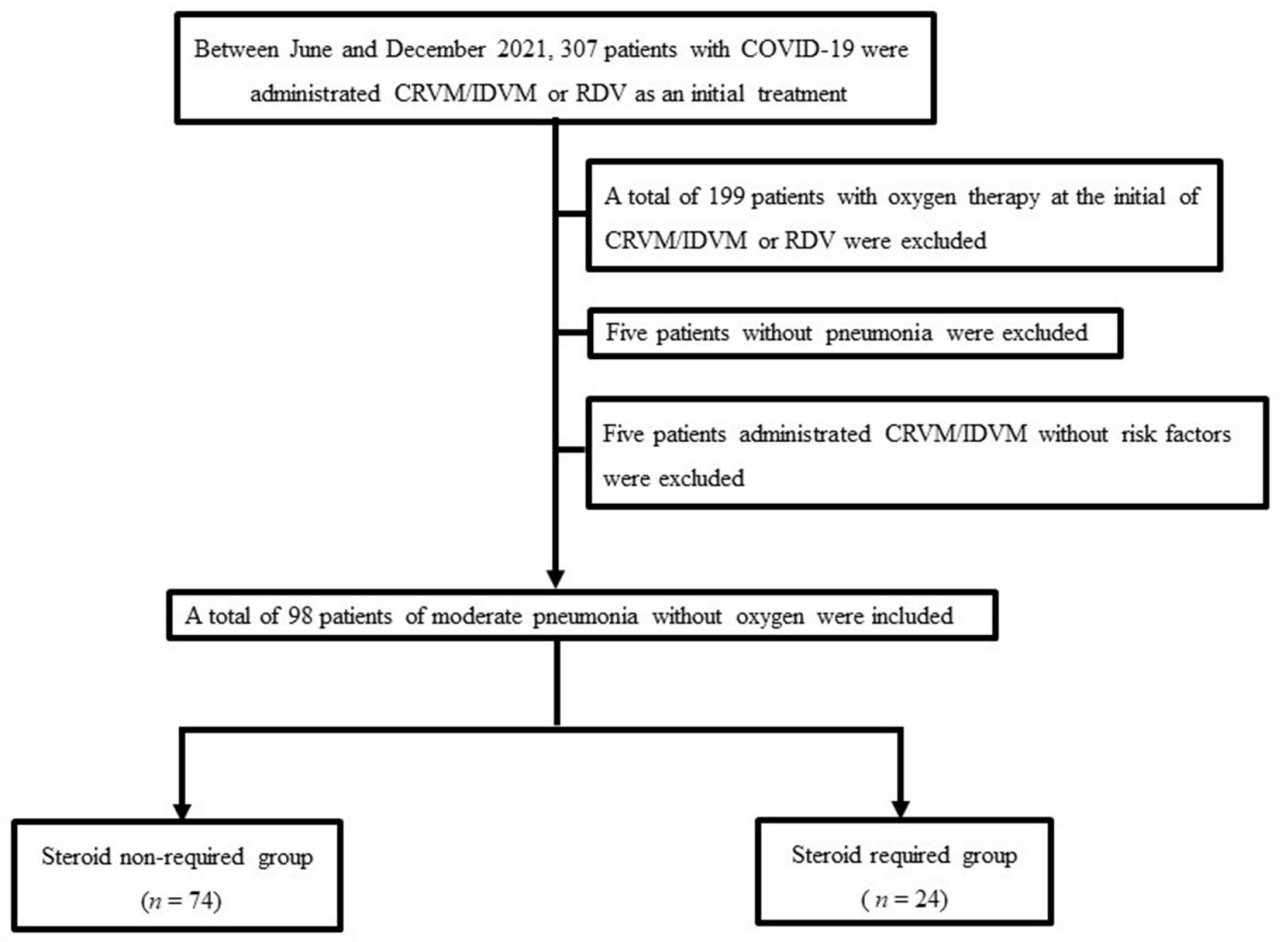
The study population flowchart. The final study cohort comprised 98 patients. COVID-19, coronavirus disease 2019. CRVM/IDVM, casirivimab/imdevimab; RDV, remdesivir.

This single-center retrospective study investigated 307 patients with COVID-19 admitted to the department of pulmonary medicine, International University of Health and Welfare Narita Hospital between June and December 2021. First, COVID-19 infections were confirmed using quantitative reverse-transcription polymerase chain reaction (RT-PCR). Then, among the 307 patients with COVID-19 admitted to our hospital during this period, 199 patients with oxygen therapy on admission were excluded, 5 patients without pneumonia were excluded, and 5 patients administered casirivimab/imdevimab (CRVM/IDVM) without risk factors were excluded. Therefore, 98 patients with COVID-19 pneumonia (without oxygen therapy) were included in our study. Of these 98 patients, the rate of Japanese population was 85.7% (*n* = 84). Subsequently, these patients were divided into the steroid-required group and the steroid non-required group. The study flowchart is shown in Figure 1.

### Clinical assessment

Data were extracted from the hospital's electronic medical records obtained from patients on hospitalization, such as their symptoms, vital signs, peripheral capillary oxygen saturation (SpO_2_), oxygen demand, laboratory test results, computed tomography (CT) scan results, and patient characteristics, including age (in years), sex, and body mass index (BMI; in kg/m^2^). These data were collected when the study's drug administration began [i.e., CRVM/IDVM or remdesivir (RDV)] (day 1). During hospitalization, vital signs were assessed every day, such as body temperature, blood pressure, pulse, oxygen saturation (SpO_2_), oxygen usage, and respiratory rate. The features of CT findings on admission were also analyzed by focusing on the presence of a bilateral shadow, subpleural shadow, ground-glass opacity, consolidation, reticulated shadow, linear shadow, interstitial thickening, pleural effusion, mediastinal lymphadenopathy, and diffuse liver concentration, in which two skilled operators (one radiologist and one pulmonologist) blinded to the clinical history subsequently classified. Finally, a chest X-ray (day 1, 4, 8, 11, 15, 29) or CT (day 1, 8, 15, 29) were mainly used to determine the deterioration of pneumonia.

### Definition of the disease severity

According to the Ministry of Health, Labor and Welfare of Japan, disease severity was categorized into four stages: mild, moderate I, moderate II, and severe. The mild disease was defined as a lack of respiratory symptoms, pneumonia, and oxygen saturation levels (SpO_2_) ≥ 96%. However, moderate disease I was defined as mild respiratory symptoms, radiological pneumonia findings, and a 93% < SpO2 < 96%. Furthermore, moderate disease II was defined as SpO2 ≤ 93% requiring oxygen support. Then, the severe disease was defined as requiring mechanical ventilation (MV) or extracorporeal membrane oxygenation (ECMO) support for ARDS (10). The diagnosis and severity were determined after discussions at the respiratory medicine conference so that the judgment would not differ depending on the clinician.

### Definition of severity risk factors in our hospital

Severity risk factors based on the Ministry of Health, Labor, and Welfare of Japan were defined as factors corresponding to any risk factor mentioned in the COVID-19 medical care guideline from Japan, the CoV-2067 trial adoption criteria, and the risk factor mentioned in the US Emergency Use Permit (EUA) (10–12). Based on these arrangements, the following factors were defined as severe factors in this study: Age ≥ 50 (years), BMI ≥ 25 kg/m^2^, diabetes mellitus, hypertension, dyslipidemia, smoking, cardiovascular disease, chronic lung disease or asthma, chronic kidney diseases, chronic liver diseases, immunosuppressive conditions, neurodevelopmental disorders, or other conditions that confer medical complexity and have medically-related technological dependence.

### Criteria and duration of casirivimab/imdevimab and remdesivir administration as a first-choice treatment

While CRVM/IDVM was administered mainly from September to December 2021, RDV was mainly administered from June to August 2021. According to a previous clinical trial, 1200 mg single intravenous infusion of CRVM/IDVM was performed for patients experiencing the severe type of COVID-19 as determined by our hospital (13). Similarly, RDV was administered intravenously for 5 days-−200 mg on the first day and 100 mg once a day after the second day of administration (14, 15). For both drugs, the observation period after administration was provided during hospitalization.

### Definition of the steroid required group

We performed a systemic corticosteroid administration when the diagnosis was considered a deterioration of pneumonia, despite initial treatment of CRVM/IDVM or RDV, defined as the steroid required group (otherwise, the steroid non-required group). This diagnosis was made when any of the following were observed: (1) pneumonia enlargement during hospitalization after chest X-ray or CT and (2) increase in oxygen demand within 24 h.

### Criteria of the H-score

The H-score demonstrates the probability of the presence of secondary haemophagocytic lymphohistiocytosis (HLH). Criteria of the H-score are shown in the Supplementary material 1. Cytopenia was defined as either a hemoglobin concentration ≤9.2 g/dL, a white blood cell count ≤5,000 leukocytes per mm3, platelet count ≤110,000 platelets per mm3, or all of these criteria combined (bone marrow is not essential to diagnose HLH). Meanwhile, immunosuppression was defined as being HIV positive or receiving long-term immunosuppressive therapies (16). Since we followed the study by Bordbar et al., zero scores were recorded for those items with no available data, such as hemophagocytosis in bone marrow smears, because it was unethical to perform a bone marrow puncture when treating COVID-19 (8).

### Statistical analysis

Summary statistics were calculated using the mean [± standard deviation (SD)], frequency distributions, or proportions for baseline variables. However, we first compared the mean values (± SD) and quartiles between the two groups for continuous variables. Subsequently, the Kolmogorov–Smirnov test (2-sided) and Shapiro–Wilk test were used to test normality, after which homoscedasticity was further tested using the F-test. Next, the Welch *t*-test and Mann–Whitney U test were performed according to the data distribution. For continuous variables, such as age and BMI, we first compared the mean values (± SD) and quartiles between the two groups. Then, Fisher's exact test was used to determine the significance of differences based on the groups. Key characteristics of the variables were later studied. In the analysis with 98 patients, a logistic regression model was fitted with age, male sex, RDV as a first-choice treatment, the corticosteroid predicting score ≥ 10 points, and IFN-λ3 levels ≥ 13.6 pg/mL. A *p* < 0.05 was considered statistically significant. Cut-off values were also evaluated using a receiver operator characteristic (ROC) curve analysis and an area under the ROC curve (AUC). Higher AUC values were considered to demonstrate better discriminatory abilities as follows: excellent discrimination, 0.9 ≤ AUC; good discrimination, 0.80 ≤ AUC < 0.90; fair discrimination, 0.70 ≤ AUC < 0.80; and poor discrimination, AUC < 0.70. For a diagnostic test to be meaningful, the AUC must be >0.5 (17, 18). Finally, all statistical analyses were conducted using EZR (Saitama Medical Center, Jichi Medical University, Saitama, Japan), a graphical user interface for R, and a modified version of the R commander designed to add statistical functions frequently used in biostatistics (19).

## Results

### Backgrounds of the patients

Table 1 shows the clinical characteristics of 98 patients in the study cohort by comparing the steroid non-required group (*n* = 74) and steroid required group (*n* = 24). No significant difference was observed between the two groups based on univariate analysis.

**Table 1:** Patient's characteristics.

**Variables**	**Corticosteroid administration**		***p*** **-Value**
	**Steroid non-required (*n* = 74)**	**Steroid required (*n* = 24)**	
Age (year)	47.0 ± 12.8	49.3 ± 12.6	0.447
Male sex (%)	51 (68.9%)	18 (75.0%)	0.619
BMI (kg/m^2^)	24.62 ± 3.86, NA = 1	26.13 ± 4.29	0.111
BMI ≥ 25 kg/m^2^ (%)	35 (47.9%), NA = 1	15 (62.5%)	0.246
Smoking (%)	36 (48.6%)	9 (37.5%)	0.359
Diabetes mellitus (%)	12 (16.2%)	3 (12.5%)	>0.999
Hypertension (%)	12 (16.2%)	5 (20.8%)	0.757
Dyslipidemia (%)	11 (14.9%)	7 (29.2%)	0.135
Malignancy (%)	2 (2.7%)	1 (4.2%)	>0.999
Chronic kidney disease (%)	1 (1.4%)	0 (0%)	>0.999
Chronic liver injury (%)	3 (4.1%)	3 (12.5%)	0.155
Chronic lung disease (%)	8 (10.8%)	2 (8.3%)	>0.999
Neuromuscular disease (%)	1 (1.4%)	1 (4.2%)	0.432
Cardiovascular disease (%)	4 (5.4%)	0 (0%)	0.569
Metabolic abnormalities (%)	1 (1%)	0 (0%)	>0.999
Number of risk factors	2.1 ± 1.3	2.3 ± 1.3	0.575
SARS-CoV-2 vaccination history	4 (5.4%)	0 (0%)	0.569
Treatments	-	-	-
CRVM/IDVM : RDV	34: 40	6: 18	0.095

*Data are presented as mean ± SD or n (%). BMI, body mass index; SARS-CoV-2, severe acute respiratory syndrome coronavirus 2; CRVM/IDVM, casirivimab/imdevimab; RDV, remdesivir*.

### Clinical parameters of the patients

Table 2 mainly shows the findings of the patient cohorts on admission, comparing the two groups. Based on the univariate analysis, although the value of SpO_2_ was significantly lower, values of the respiratory rate, aspartate transaminase (AST), ferritin, lactate dehydrogenase (LDH), interferon lambda 3 (IFN-λ3), and the H-score were significantly higher in the steroid required group than in the steroid non-required group. Additionally, the ratio of the body temperature (BT) ≥ 38.5°C was higher in the treatment failure group than in the treatment success group (62.5 *vs*. 37.8%). Still, it did not lead to a statistically significant difference (*p* = 0.0569). Meanwhile, no significant difference was observed between the two groups in analyzing CT findings.

**Table 2:** Characteristics and outcomes of patients on admission.

**Variables**	**Steroid administration**		***p*** **-Value**
	**Steroid non-required (*n* = 74)**	**Steroid required (*n* = 24)**	
Duration from symptom onset to treatment initiation (days)	6.8 ± 2.8, NA = 1	6.0 ± 2.5	0.2450
Body temperature (°C)	38.2 ± 1.0	38.5 ± 0.8	0.0954
Body temperature ≥ 38.5 (°C)	28 (37.8%)	15 (62.5 %)	0.0569
Respiratory rate (breaths/min)	20.1 ± 2.7, NA = 1	21.8 ± 3.6, NA = 1	0.0212
SpO_2_ (%)	95.6 ± 1.4	94.9 ± 1.7	0.0267
Dyspnea or shortness of breath (%)	24 (32.4%)	8 (33.3%)	>0.9999
**Laboratory findings**			
T-Bil (mg/dL)	0.7 ± 0.3	0.7 ± 0.3	0.5360
AST (U/L)	44.2 ± 30.0	82.4 ± 74.7	0.0219
ALT (U/L)	46.1 ± 46.0, NA = 1	86.5 ± 95.8	0.0566
γGTP (U/L)	80.9 ± 87.6	142.6 ± 151.2	0.0681
Ferritin (ng/mL)	583.5 ± 550.0, NA = 3	1,093.1 ± 939.9	0.0178
TG (mg/dL)	128.5 ± 71.5, NA = 1	141.8 ± 90.2, NA = 1	0.4700
LDH (U/L)	303.5 ± 93.0	384.2 ± 116.6	<0.0001
CRP (mg/dL)	4.32 ± 3.70	5.48 ± 4.26	0.2000
PCT (ng/mL)	0.09 ± 0.08, NA = 3	0.13 ± 0.11, NA = 1	0.1040
IFN-λ3 (pg/mL)	12.0 ± 9.9, NA = 3	23.6 ± 18.3, NA = 1	0.0071
Hb (g/dL)	14.7 ± 1.7	14.8 ± 2.0	0.8130
WBC (× 1,000/μL)	4.94 ± 1.80	4.69 ± 1.81	0.5520
Plt (× 1,000/μL)	195.9 ± 64.8	152.8 ± 52.9	0.0040
D-dimer (μg/mL)	0.82 ± 0.69	0.89 ± 0.63	0.6490
Fibrinogen (mg/dL)	474.7 ± 90.5 (NA = 33)	488.2 ± 134.1 (NA = 6)	0.6990
**H-score**	47.8 ± 30.1	62.8 ± 31.1	0.0378
**CT findings**			
Bilateral shadows (%)	66 (89.2%)	24 (100%)	0.1930
Subpleural shadows (%)	52 (70.3%)	17 (70.8%)	>0.9999
Ground-glass opacity (%)	70 (94.6%)	24 (100%)	0.5690
Consolidation (%)	17 (23.0%)	6 (25.0%)	>0.9999
Reticulated shadows (%)	2 (2.7%)	1 (4.2%)	>0.9999
Linear shadows (%)	7 (9.5%)	1 (4.2%)	0.6750
Interstitial thickening (%)	18 (24.3%)	7 (29.2%)	0.7880
Hepatomegaly (%)	2 (2.7%)	0 (0%)	>0.9999
Splenomegaly (%)	7 (9.5%)	0 (0%)	0.1890

*Data are presented as mean ± SD or n (%). T-Bil, total bilirubin; AST, aspartate transaminase; ALT, alanine transaminase; γGTP, γ-glutamyltranspeptidase; TG, triglyceride; LDH, lactate dehydrogenase; CRP, C-reactive protein; PCT, procalcitonin; IFN-λ3, interferon lambda 3; Hb, hemoglobin; WBC, white blood cell; Plt, platelet*.

### The setting and result of the predicting score model for predicting the administration of corticosteroids (a SP-score)

**Figure 2:**
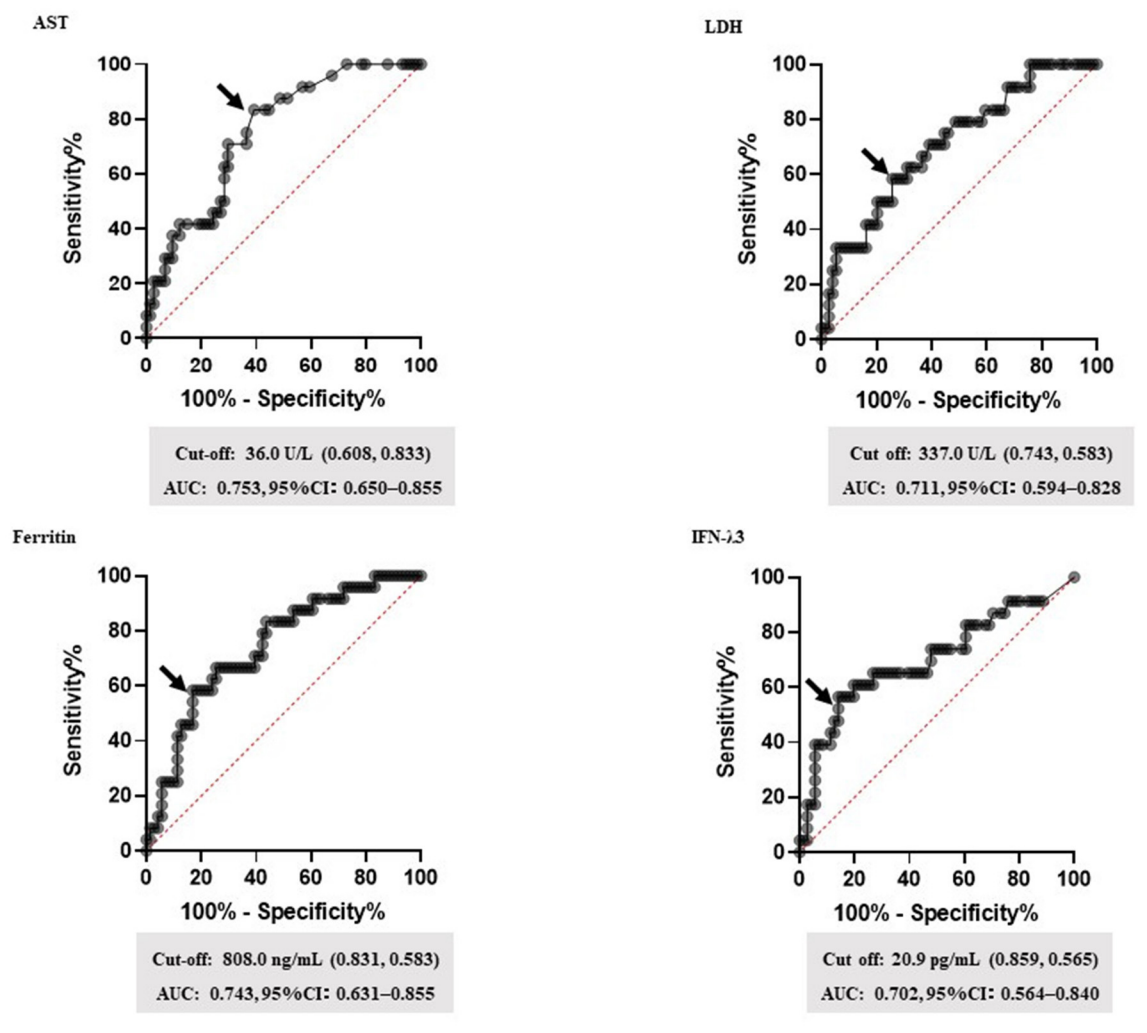
The setting of biomarker cut-off values. Receiver operating characteristic (ROC) curves for the highest area under the curve (AUC) values are shown. ROC curves were analyzed to determine the cut-off values for each biomarker. The arrow indicates the cut-off point for each factor. Cut-off values (specificity and sensitivity), AUCs, and 95% confidence interval (CI) for each biomarker are shown. AST, aspartate transaminase; LDH, lactate dehydrogenase; IFN-λ3, interferon lambda 3.

Based on the data, corticosteroid requirement score was established. Table 3 shows the predicting score model for corticosteroid administration (a steroid predicting score; SP-score). Score parameters were constructed based on the following: (1) the duration of symptom onset to treatment initiation ≥ 7 d, (2) respiratory rate ≥ 22 breaths/min, (3) SpO_2_ ≤ 95%, (4) BT ≥ 38.5°C, (5) AST ≥ 40 U/L, (6) LDH ≥ 340 U/L, (7) ferritin ≥ 800 ng/mL, and (8) IFN-λ3 ≥ 20 pg/mL. Subsequently, while the cut-off values of AST, LDH, ferritin, and IFN-λ3 were determined based on ROC analysis (Figure 2), cut-off values of the respiratory rate and SpO_2_ were determined based on univariate analysis (Table 2).

**Table 3:** A scoring model for predicting the administration of corticosteroids.

**Components**	**Number of points**
Duration from symptom onset to treatment initiation ≥ 7 days	1
Respiratory rate ≥ 22 breaths/min	4
SpO_2_ ≤ 95%	4
Body temperature ≥ 38.5 (°C)	2
AST ≥ 40 U/L	3
LDH ≥ 340 U/L	3
Ferritin ≥ 800 ng/mL	3
IFN-λ3 ≥ 20 pg/mL	3

*AST, aspartate transaminase; LDH, lactate dehydrogenase; IFN-λ3, interferon lambda 3*.

Given that vital signs are one of the most important findings that reflect the general condition, we added four points if it corresponded to the respiratory rate ≥ 22 breaths/min or SpO_2_ ≤ 95%. The frequency of steroids-required group with BT ≥ 38.5°C tended to be (not statistically) higher than that of the steroid non-required group; thus, we added two points if it corresponded to the BT ≥ 38.5°C. Furthermore, three points were added if they corresponded to the AST, LDH, ferritin, or IFN-λ3, and one point was added based on the duration of symptom onset to treatment initiation ≥ 7 d. The total score was defined as the predicting score of a patient.

### Setting of the cut-off value of SP-score

**Figure 3:**
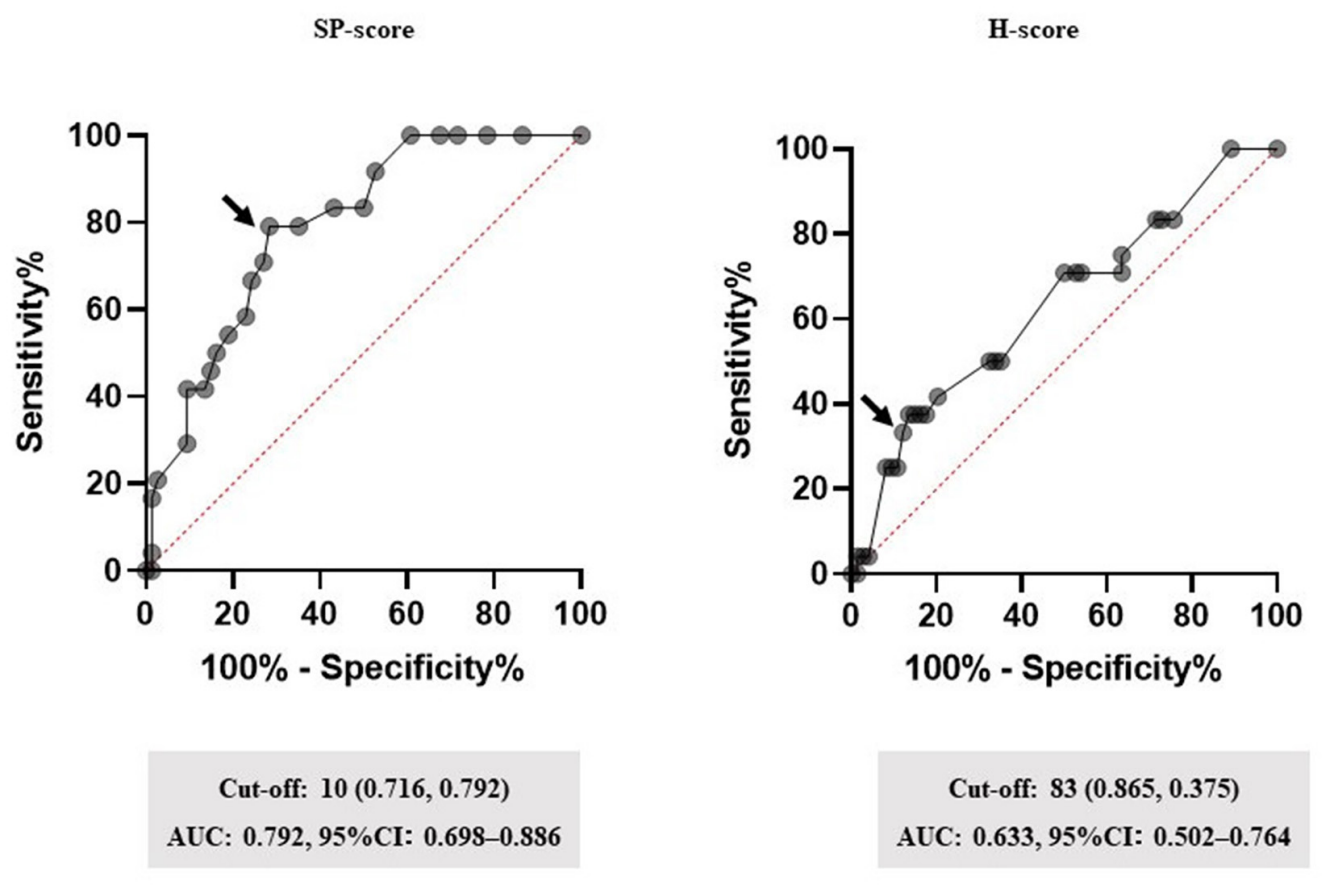
Performance of the SP-score. Receiver operating characteristic (ROC) curves for the highest area under the curve (AUC) values are shown. Subsequently, ROC curves were analyzed to determine the cut-off values for each biomarker. The arrow indicates the cut-off point for each factor. Cut-off values (specificity and sensitivity), AUCs, and 95% confidence interval (CI) for each biomarker are shown. SP-score, steroid predicting score.

Figure 3 shows the performance of the SP-score, compared with the H-score. ROC analysis defined the cut-off values of the SP-score and the H-score, both of which were predicted to account for corticosteroid administration. The cut-off value of the SP-score was 10 (AUC: 0.792; a fair discrimination, 95%CI: 0.698–0.886). Furthermore, although the H-score >169 was 93% sensitive and 86% specific for HLH, the score was not reported to be as high as HLH in patients with COVID-19 (8). Additionally, from the result of the univariate analysis (Table 2), the H-score of the steroid-required group presented significantly higher scores than the steroid non-required group (62.8 ± 31.1 *vs*. 47.8 ± 30.1, *p* = 0.0378). Besides, none of the patients in this study demonstrated a score of 169 or more. Therefore, the cut-off value of the H-score for predicting steroid administration was modified using ROC analysis. As a result, the modified cut-off value of the H-score was 83 (AUC: 0.633; a poor discrimination, 95%CI: 0.502–0.764). Comparing the AUC of the SP-score and the H-score, the AUC of the SP-score was significantly higher than that of the H-score (*p* = 0.0241).

### Multivariate logistic regression analysis of factors for steroid administration

Table 4 shows the analysis of factors accounting for steroid administration. Factors like age, male sex, RDV as a first-choice treatment, a SP-score ≥ 10 points, and IFN-λ3 ≥ 13.6 pg/mL were analyzed. Among these factors, the multivariate analysis showed that a SP score ≥ 10 points significantly affected the steroid administration (OR: 6.91, 95%CI: 2.120–22.500, *p* = 0.0014).

**Table 4:** Multivariate logistic regression analysis of factors accounting for conditions requiring steroid administration.

**Variables**	**OR**	**95%CI**	***p*** **-value**
Age (year)	1.02	0.967–1.080	0.4470
Male sex	1.24	0.314–4.900	0.7590
RDV as a first-choice treatment	3.11	0.913–10.600	0.0696
Steroid predicting score ≥ 10 (points)	6.91	2.120–22.500	0.0014
IFN-λ3 ≥ 13.6 pg/mL	3.14	0.971–10.200	0.0560

*The multivariate logistic regression analysis of factors accounting for steroid administration. Factors like age, male sex, RDV as a first-choice treatment, a steroid predicting score ≥ 10 points, and IFN-λ3 ≥ 13.6 pg/mL were adjusted. Although these factors were adjusted, the analysis showed that a steroid predicting score ≥ 10 points significantly affected the steroid administration. RDV, remdesivir; IFN-λ3, interferon lambda 3; OR, odds ratio; 95% CI, 95% confidence interval*.

## Discussion

This study revealed several findings. First, a score for the early predictor of COVID-19 hyper-inflammation status named the steroid predicting score (SP-score), composed of a body temperature ≥ 38.5°C, respiratory rate ≥ 22 breaths/min, SpO_2_ ≤ 95%, a duration from symptom onset to treatment initiation ≥ 7 d, AST ≥ 40 U/L, LDH ≥ 340 U/L, Ferritin ≥ 800 ng/mL, and IFN-λ3 ≥ 20 pg/mL, showed good AUC values in the ROC analysis. Second, comparing the H-score and SP-score with ROC analysis, the SP-score showed a significantly higher AUC score than the H-score (Figure 2). Third, compared with IFN-λ3 ≥ 13.6 pg/mL, a SP-score ≥ 10 was proposed to affect the steroid administration condition (Table 4).

The timing of administering corticosteroids is one of the key points for improving the pathophysiology of COVID-19. In a previous RECOVERY Trial, the mortality benefit of corticosteroid therapy in COVID-19 was only evident in those with a symptom duration of seven days or more (6, 20). However, a higher death rate in the dexamethasone group was observed in patients with a symptom duration of fewer than seven days (20). Thus, considering the importance of corticosteroid administration timing, we evaluated the duration from symptom onset to treatment initiation as a part of the corticosteroid predicting score. In another Metcovid trial, a trend toward increased mortality was observed in patients under 60 years of age, who had lower C-reactive protein (CRP) levels, indicating poorer inflammatory status and worse disease severity (21). In our study, a SP-score ≥ 10 significantly accounted for the condition requiring steroids, that is, a condition suggesting hyper-inflammation. The results propose that scores may be a useful tool for predicting the most appropriate timing of steroid administration.

Another advantage of the SP-score is that it can be used to determine hospitalization indications for patients with COVID-19. There is a lack of safety and efficacy data on the use of corticosteroids in COVID-19 outpatients, the National Institutes of Health (NIH)-issued COVID-19 treatment guidelines recommend against the use of corticosteroids to treat outpatients (22). Furthermore, there is no evidence to support corticosteroid use in patients with COVID-19 who do not receive respiratory support (6, 20). Therefore, given that corticosteroids are often used in clinical practice, the SP-score may be useful as a criterion for hospitalization.

Interferon is a type of cytokine, divided into type I (IFN-α, β), type II (IFN-γ), and type III (IFN-λs). It is an inhibitor of viral infection as an innate immune system response, functioning as the first line of host defense against pathogens, including SARS-CoV-2 (23–25). In the innate immune system, dendritic cells (DCs) and macrophage populations are highly sensitive to interferon lambdas (IFN-λs) (26, 27). Specifically, interferon lambdas (IFN-λs) are a family of innate immune cytokines composed of IFN-λs 1–4 that are critical mediators of barrier immunity (27). Furthermore, Sugiyama et al. previously demonstrated IFN-λ3 as a predictive biomarker for severe onset (28). It has also been reported that IFN-λ3 concentrations in the serum increase in COVID-19 patients a few days before oxygen administration. Therefore, this report proposes that the rise in IFN-λ3 can be a biomarker that predicts the severity of COVID-19 in the relatively early phase of the disease. Indeed, IFN-λ3 is hypothesized to affect hyper-inflammatory conditions from the result of the univariate analysis. However, in the multivariate analysis, a cut-off value of the SP-score ≥ 10 significantly affected the hyper-inflammation status compared with IFN-λ3 ≥ 13.6 pg/mL, proposing the steroid predicting score as an independent hyper-inflammation predictor.

Comparing the H-score and SP-score parameters, both are similar in that they contain fever, AST, and ferritin levels. While several negative views on the usefulness of the H-score as a predictor of severe COVID-19 exist, opinions that the H-score can be useful if modified have also been reported (8). For example, Bordbar et al. proposed that a higher H-score was associated with more ICU admissions, extended hospitalization periods, and a higher mortality rate (8). Furthermore, the H-score with a new cut-off is considered more practical in predicting disease severity in patients with severe COVID-19. Besides, by analyzing H-score parameters, Gürsoy et al. proposed that although COVID-19 pneumonia had similar findings to hyper-inflammatory syndromes, these findings did not have typical features like in MAS/Shlh cytokine storm development (7). Based on our findings, the SP-score showed a higher AUC value than the H-score, indicating high accuracy in predicting high-inflammatory conditions, such as “cytokine storms.”

Our study has several limitations. First, this study was a single-center study. Second, the overall sample size in the final study cohort as well as the sample size in the steroid required group was limited. However, it was necessary to unify the patient background including virus variant and therapeutic drugs in order to compare non-required group and steroid required group. In Japan, the delta variant was the mainstream from June 2021 to December 2021 (29). During this period, RDV was recommended as treatments for moderate disease, and IDVM / CRVM was additionally approved for the disease in July 2021 (30, 31). Thus, as an initial treatment for moderate disease I, while CRVM/IDVM was administered mainly from September to December 2021, RDV was mainly administered from June to August 2021. Because there is no large-scale study that directly compares the effects of these two drugs, it was necessary to show in the present study that there was no statistically significant difference in the frequency of steroid administration between RDV and CRVM/IDVM (as shown in Tables 1, 4). To confirm the accuracy of SP-score with various treatments and other virus variants, further studies with a larger sample size are needed. Third, as shown in Table 4, the SP-score≥10 points indicated a wide confidence interval in multivariate analysis. One of the reasons for this result may be the small sample size. For the SP-score to become widely used in clinical practice, larger scale studies are desired.

Fourth, the racial of the analyzed patients was not identical. Of the 98 all patients analyzed in this study, 84 were Japanese. However, we also conducted an analysis of Japanese limited population (as shown in Supplementary Tables and Figures). Comparing Japanese limited population with all patients, most of the results were similar except for multivariate analyses of factors accounting for steroid administration. In the analysis of all patients, only SP-score was the independent factor affecting steroid administration. On the other hand, in the Japanese limited population analysis, both SP-score and IFN-λ3 was the independent factor affecting steroid administration. However, In both of these analyzes, the SP-score had the highest odds ratio. Moreover, the elevated SP-score showed a higher odds ratio than the elevated IFN-λ3 even in the analysis of Japanese limited population. This result may suggest that, in clinical practice, it is important to comprehensively evaluate not only biomarkers, but also symptoms, clinical course, vital signs, and inflammatory markers. Fifth, of all the patients enrolled in this study, the Japanese population accounted for 85.7%. Thus, the our study findings may not be generisable to other populations in different countries due to underlying ethnic differences. Further studies would be required in different countries to confirm generalisability of findings from this study. Sixth, in this study, H-score was evaluated as an optimal steroid administration. A previous report concludes that modified H-score with a new cut-off seems to predict disease severity in patients with severe COVID-19 (not the timing of steroid administration). However, the present study suggests that the SP-score may be better than the H-score as a score that predicts the appropriate timing of steroid administration.

In conclusion, the SP-score may be useful in pre-empting hyper-inflammation. Furthermore, the SP-score may be better than the H-score as a clinical score that predicts the appropriate timing of steroid administration. Moreover, the SP-score may be useful as a criterion for hospitalization. The data may provide important information to facilitate a prospective study on a larger scale in this field.

## Data availability statement

The raw data supporting the conclusions of this article will be made available by the authors, without undue reservation.

## Ethics statement

All study procedures were conducted according to the standards of the Ethical Review Board of the International University of Health and Welfare (approval number 20-Nr-101; 2021/02/22 approved) and conformed to the 1964 Declaration of Helsinki and its subsequent amendments or comparable ethical standards. Written informed consent from the participants' legal guardian/next of kin was not required to participate in this study in accordance with the national legislation and the institutional requirements.

## Author contributions

YTak and KT: study conception, design, and drafting of the manuscript. YTak, YH, TKinos, HT, KK, TKinou, YI, YS, AF, and YTad: data collection, analysis, and review. YTak, JT, YH, TKinos, HT, KK, TKinou, YI, YS, AF, YK, YTo, YTad, and KT: statistical analysis and interpretation. JT, YTo, YK, and KT: administrative and technical support. JT, YH, TKinos, HT, KK, TKinou, YI, YS, AF, YK, YTo, YTad, and KT: critical revision of the manuscript. All authors contributed to the article and approved the submitted version.

## Funding

A grant from the International University of Health and Welfare (IUHW research grant 2020) was used to support this research.

## Conflict of interest

JT received research funding from Teijin Pharma Ltd (a collaborative research project with Chiba University and Teijin Pharma Ltd). YTo received lecture fees from GlaxoSmithKline, AstraZeneca, and Novartis Pharma. The remaining authors declare that the research was conducted in the absence of any commercial or financial relationships that could be construed as a potential conflict of interest.

## Publisher's note

All claims expressed in this article are solely those of the authors and do not necessarily represent those of their affiliated organizations, or those of the publisher, the editors and the reviewers. Any product that may be evaluated in this article, or claim that may be made by its manufacturer, is not guaranteed or endorsed by the publisher.

## Supplementary material

Supplementary Figure 1 The setting of biomarker cut-off values by analysing japanese patients. Receiver operating characteristic (ROC) curves for the highest area under the curve (AUC) values are shown. ROC curves were analyzed to determine the cut-off values for each biomarker. The arrow indicates the cut-off point for each factor. Cut-off values (specificity and sensitivity), AUCs, and 95% confidence interval (CI) for each biomarker are shown. AST, aspartate transaminase; LDH, lactate dehydrogenase; IFN-λ3, interferon lambda 3.

Supplementary Figure 2 Performance of the SP-score by analysing japanese patients. Receiver operating characteristic (ROC) curves for the highest area under the curve (AUC) values are shown. Subsequently, ROC curves were analyzed to determine the cut-off values for each biomarker. The arrow indicates the cut-off point for each factor. Cut-off values (specificity and sensitivity), AUCs, and 95% confidence interval (CI) for each biomarker are shown. SP-score, steroid predicting score.

Supplementary Table 1 The H-score criteria.

Supplementary Table 2 Japanese patient's characteristics.

Supplementary Table 3 Characteristics and outcomes of japanese patients on admission.

Supplementary Table 4 Multivariate logistic regression analysis of factors accounting for conditions requiring steroid administration; japanese limited data (*n* = 84).
