# Supplementary figures and images for: Development of a novel score model to predict hyperinflammation in COVID-19 as a forecast of optimal steroid administration timing
Source: Front Med (Lausanne). 2022 Aug 9;9:935255. doi: 10.3389/fmed.2022.935255 (PMC9395649; doi:10.3389/fmed.2022.935255)

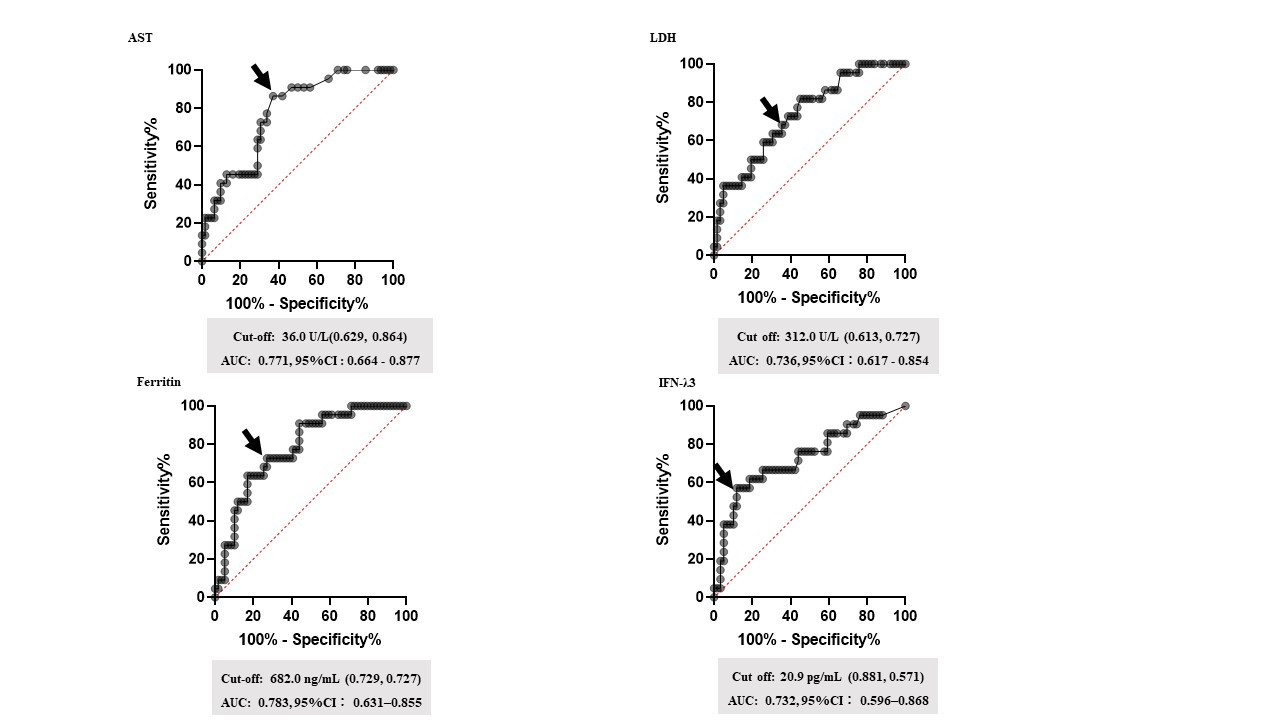

Supplement: Supplementary Figure 1 — The setting of biomarker cut-off values by analysing japanese patients. Receiver operating characteristic (ROC) curves for the highest area under the curve (AUC) values are shown. ROC curves were analyzed to determine the cut-off values for each biomarker. The arrow indicates the cut-off point for each factor. Cut-off values (specificity and sensitivity), AUCs, and 95% confidence interval (CI) for each biomarker are shown. AST, aspartate transaminase; LDH, lactate dehydrogenase; IFN-λ3, interferon lambda 3. [file Image_1.JPEG]

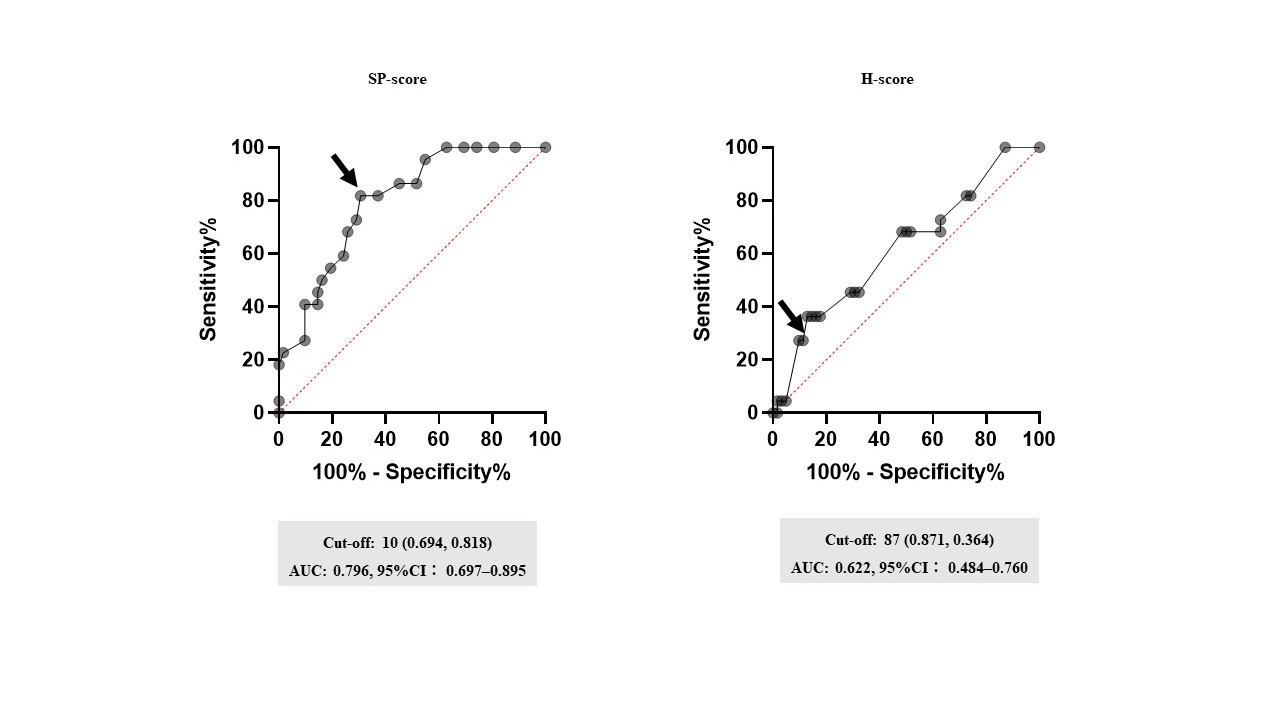

Supplement: Supplementary Figure 2 — Performance of the SP-score by analysing japanese patients. Receiver operating characteristic (ROC) curves for the highest area under the curve (AUC) values are shown. Subsequently, ROC curves were analyzed to determine the cut-off values for each biomarker. The arrow indicates the cut-off point for each factor. Cut-off values (specificity and sensitivity), AUCs, and 95% confidence interval (CI) for each biomarker are shown. SP-score, steroid predicting score. [file Image_2.JPEG]
