# Supplementary material for: Development of a novel score model to predict hyperinflammation in COVID-19 as a forecast of optimal steroid administration timing
Source: Front Med (Lausanne). 2022 Aug 9;9:935255. doi: 10.3389/fmed.2022.935255 (PMC9395649; doi:10.3389/fmed.2022.935255)
Supplement: Supplementary Table 1 — The H-score criteria. [file Table_1.DOCX]

**Supplement table 1–The H-score criteria**

| **Components** | **Number of scores** |
| --- | --- |
| **Temperature (°C)** |  |
| <38.4 | 0 |
| 38.4–39.4 | 33 |
| >39.4 | 49 |
| **Presence of organomegaly** |  |
| None | 0 |
| Hepatomegaly or splenomegaly | 23 |
| Hepatomegaly and splenomegaly | 38 |
| **Number of cytopenias** |  |
| 1 lineage | 0 |
| 2 lineages | 24 |
| 3 lineages | 34 |
| **Ferritin levels (ng/mL)** |  |
| <2000 | 0 |
| 2000–6000 | 35 |
| >6000 | 50 |
| **TG (mg/dl)** |  |
| <132.7 | 0 |
| 132.7–354 | 44 |
| >354 | 64 |
| **Fibrinogen levels (mg/dl)** |  |
| >250 | 0 |
| ≤250 | 30 |
| **AST levels (U/L)** |  |
| <30 | 0 |
| ≥30 | 19 |
| **Hemophagocytosis features on bone marrow aspirate** |  |
| No | 0 |
| Yes | 35 |
| **Known immunosuppression** |  |
| No | 0 |
| Yes | 18 |

TG, triglyceride; AST, aspartate transaminase.
