# Supplementary material for: Development of a novel score model to predict hyperinflammation in COVID-19 as a forecast of optimal steroid administration timing
Source: Front Med (Lausanne). 2022 Aug 9;9:935255. doi: 10.3389/fmed.2022.935255 (PMC9395649; doi:10.3389/fmed.2022.935255)
Supplement: Supplementary Table 2 — Japanese patient's characteristics. [file Table_2.DOCX]

**Supplement table 2– Japanese patient’s Characteristics**

| **Variables** | **Corticosteroid administration** | | ***p*-value** |
| --- | --- | --- | --- |
|  | **Steroid non-required (n = 62)** | **Steroid required (n = 22)** |  |
| Age (year) | 48.4 ± 12.8 | 50.4±12.4 | 0.520 |
| Male sex (%) | 43 (69.3%) | 17 (77.3%) | 0.589 |
| BMI (kg/m^2^) | 24.39 ± 3.87 | 25.9 ± 4.32 | 0.139 |
| BMI ≥ 25 kg/m^2^ (%) | 27 (44.3%), NA=1 | 13 (59.1%) | 0.320 |
| Smoking (%) | 31 (50.0%) | 9 (40.9%) | 0.620 |
| Diabetes mellitus (%) | 11 (17.7%) | 2 (9.1%) | 0.498 |
| Hypertension (%) | 11 (17.7%) | 5 (22.7%) | 0.753 |
| Dyslipidemia (%) | 11 (17.7%) | 7 (31.2%) | 0.226 |
| Malignancy (%) | 2 (3.2%) | 1 (4.5%) | >0.999 |
| Chronic kidney disease (%) | 1 (1.6%) | 0 (0%) | >0.999 |
| Chronic liver injury (%) | 3 (4.8%) | 3 (13.6%) | 0.182 |
| Chronic lung disease (%) | 8 (12.9%) | 2 (9.1%) | >0.999 |
| Neuromuscular disease (%) | 1 (1.6%) | 1 (4.5%) | 0.458 |
| Cardiovascular disease (%) | 4 (6.5%) | 0 (0%) | 0.569 |
| Metabolic abnormalities (%) | 1 (1.6%) | 0 (0%) | >0.999 |
| Number of risk factors | 2.3 ± 1.3 | 2.4 ± 1.3 | 0.743 |
| SARS-CoV-2 vaccination history | 4 (6.5%) | 0 (0%) | 0.569 |
| Treatments | - | - | - |
| CRVM/IDVM : RDV | 26 : 36 | 5 : 17 | 0.129 |

Data are presented as mean ± SD or n (%). BMI, body mass index; SARS-CoV-2, severe acute respiratory syndrome coronavirus 2; CRVM/IDVM, casirivimab/imdevimab; RDV, remdesivir.
