# Supplementary material for: Development of a novel score model to predict hyperinflammation in COVID-19 as a forecast of optimal steroid administration timing
Source: Front Med (Lausanne). 2022 Aug 9;9:935255. doi: 10.3389/fmed.2022.935255 (PMC9395649; doi:10.3389/fmed.2022.935255)
Supplement: Supplementary Table 3 — Characteristics and outcomes of japanese patients on admission. [file Table_3.DOCX]

**Supplement table 3– Characteristics and outcomes of japanese patients on admission**

| **Variables** | **Steroid administration** | | ***p*-value** |
| --- | --- | --- | --- |
|  | **Steroid non-required (*n* = 62)** | **Steroid required(*n* = 22)** |  |
| Duration from symptom onset to treatment initiation (days) | 6.8 ± 2.7, NA = 1 | 5.8 ± 2.4 | 0.1070 |
| Body temperature (°C) | 38.1 ± 1.0 | 38.5 ± 0.7 | 0.0646 |
| Body temperature ≥ 38.5 (°C) | 25 (40.3%) | 14 (63.6 %) | 0.0820 |
| Respiratory rate (breaths/min) | 20.1 ± 2.9, NA = 1 | 22.0 ± 3.6, NA = 1 | 0.0157 |
| SpO_2_ (%) | 95.6 ± 1.4 | 95.0 ± 1.6 | 0.1320 |
| Dyspnea or shortness of breath (%) | 20 (32.3%) | 7 (31.8%) | >0.9999 |
| **Laboratory findings** |  |  |  |
| T-Bil (mg/dL) | 0.7 ± 0.3 | 0.7 ± 0.3 | 0.6990 |
| AST (U/L) | 43.5 ± 28.3 | 86.0± 77.0 | 0.0188 |
| ALT (U/L) | 44.1± 39.1, NA = 1 | 90.2 ± 99.3 | 0.0450 |
| γGTP (U/L) | 85.8 ± 93.1 | 151.7 ± 155.0 | 0.0712 |
| Ferritin (ng/mL) | 584.1± 558.1, NA = 3 | 1170.2 ± 944.5 | 0.0109 |
| TG (mg/dL) | 125.3 ± 57.3, NA = 1 | 125.2 ± 46.5, NA = 1 | 0.9960 |
| LDH (U/L) | 303.4 ± 90.6 | 390.1± 118.2 | <0.0001 |
| CRP (mg/dL) | 4.45 ± 3.88 | 5.64 ± 4.40 | 0.2370 |
| PCT (ng/mL) | 0.08 ± 0.07, NA = 3 | 0.14± 0.12, NA = 1 | 0.0557 |
| IFN-λ3 (pg/ml) | 11.5 ± 9.7, NA = 3 | 24.4± 18.6, NA = 1 | 0.0058 |
| Hb (g/dL) | 14.7 ± 1.8 | 14.8 ± 2.1 | 0.7730 |
| WBC (×1000/μL) | 4.97 ± 1.82 | 4.58 ± 1.83 | 0.3960 |
| Plt (×1000/μL) | 195.6 ± 63.1 | 151.4 ± 52.3 | 0.0042 |
| D-dimer (μg/mL) | 0.86 ± 0.74 | 0.92 ± 0.65 | 0.7310 |
| Fibrinogen (mg/dL) | 474.7 ± 90.5 (NA = 29) | 488.2 ± 134.1 (NA = 6) | 0.5670 |
| **H-score** | 47.0 ± 31.1 | 61.6 ± 32.2 | 0.0640 |
| **CT findings** |  |  |  |
| Bilateral shadows (%) | 54 (87.1%) | 22 (100%) | 0.1040 |
| Subpleural shadows (%) | 44 (71.0%) | 15 (68.2%) | 0.7930 |
| Ground-glass opacity (%) | 59 (95.2%) | 22 (100%) | 0.5630 |
| Consolidation (%) | 16 (25.8%) | 6 (27.3%) | >0.9999 |
| Reticulated shadows (%) | 2 (3.2%) | 1 (4.5%) | >0.9999 |
| Linear shadows (%) | 7 (11.3%) | 1 (4.5%) | 0.6740 |
| Interstitial thickening (%) | 13 (21.0%) | 6 (27.3) | 0.5620 |
| Hepatomegaly (%) | 1 (1.6%) | 0 (0%) | >0.9999 |
| Splenomegaly (%) | 5 (8.1%) | 0 (0%) | 0.3190 |

Data are presented as mean ± SD or n (%). T-Bil, total bilirubin; AST, aspartate transaminase; ALT, alanine transaminase; γGTP, γ-glutamyltranspeptidase; TG, triglyceride; LDH, lactate dehydrogenase; CRP, C-reactive protein; PCT, procalcitonin; IFN-λ3, interferon lambda 3; Hb, hemoglobin; WBC, white blood cell; Plt, platelet.
