# Supplementary material for: Development of a novel score model to predict hyperinflammation in COVID-19 as a forecast of optimal steroid administration timing
Source: Front Med (Lausanne). 2022 Aug 9;9:935255. doi: 10.3389/fmed.2022.935255 (PMC9395649; doi:10.3389/fmed.2022.935255)
Supplement: Supplementary Table 4 — Multivariate logistic regression analysis of factors accounting for conditions requiring steroid administration; japanese limited data (n = 84). [file Table_4.DOCX]

**Supplement table 4– Multivariate logistic regression analysis of factors accounting for conditions requiring steroid administration; japanese limited data (n=84)**

| **Variables** | **OR** | **95%CI** | ***p-*value** |
| --- | --- | --- | --- |
| Age (year) | 1.03 | 0.967–1.100 | 0.3710 |
| Male sex | 1.85 | 0.392–8.720 | 0.4370 |
| RDV as a first-choice treatment | 3.41 | 0.875–13.300 | 0.0771 |
| Steroid predicting score ≥ 10 (points) | 7.55 | 2.070–27.500 | 0.0022 |
| IFN-λ3 ≥ 13.6 pg/mL | 4.30 | 1.200–15.400 | 0.0251 |

The multivariate logistic regression analysis of factors accounting for steroid administration. Factors like age, male sex, RDV as a first-choice treatment, a steroid predicting score ≥ 10 points, and IFN-λ3 ≥ 13.6 pg/mL were adjusted. Among these factors, the analysis showed that a steroid predicting score ≥ 10 points and IFN-λ3 ≥ 13.6 pg/mL significantly affected the steroid administration. RDV, remdesivir; IFN-λ3, interferon lambda 3; OR, odds ratio; 95% CI, 95% confidence interval.
